# Accidental exposure to gas emissions from transit goods treated for pest control

**DOI:** 10.1186/1476-069X-13-110

**Published:** 2014-12-13

**Authors:** Stefan Kloth, Xaver Baur, Thomas Göen, Lygia Therese Budnik

**Affiliations:** Division of Occupational Toxicology and Immunology, Institute for Occupational and Maritime Medicine (ZfAM), University Medical Center Hamburg-Eppendorf, University of Hamburg, Marckmannstrasse 129 b, Bld. 3, 20539 Hamburg, Germany; Institute for Occupational Medicine, Campus Benjamin Franklin, Charité-School of Medicine, Berlin, Germany; Institute and Outpatient Clinic of Occupational, Social and Environmental Medicine, Friedrich-Alexander-University Erlangen-Nürnberg, Erlangen, Germany; Robert Koch Institute, Unit Strengthening Global Biosecurity, Berlin, Germany

**Keywords:** Fumigants, Methyl bromide, Ethylene oxide, Hemoglobin adducts, Past exposure, Transit goods and production parts, Off gassing, Human biomonitoring

## Abstract

**Background:**

International phytosanitary standards ISPM 15 require (since 2007) fumigation or heat treatment for shipping and storage. Those dealing with fumigated freight might be accidentally exposed. In this paper we report a series of three accidents of six storage room workers in a medium sized company regularly importing electronic production parts from abroad.

**Methods:**

Patients (n = 6, aged from 32–54 yrs.) and control group (n = 30, mean 40 yrs.) donated blood and urine samples. The fumigants: ethylene oxide, methyl bromide, chloropicrin, ethylene dichloride, other halo-alkanes and solvents were analyzed by headspace gas chromatography/mass spectrometry (GCMS). For the quantitation of long term exposure/s, macromolecular reaction products (hemoglobin adducts) were used (with GCMS) as molecular dosimeter; additionally 8-OHdG and circulating mtDNA (cmtDNA) were analyzed as nonspecific biological effect markers.

**Results:**

The hemoglobin adducts N-methyl valine (MEV) and N-(2-hydroxy ethyl) valine (HEV) were elevated after exposure to the alkylating chemicals methyl bromide and ethylene oxide. Under the consideration of known elimination kinetics and the individual smoking status (biomonitored with nicotine metabolite cotinine and tobacco specific hemoglobin adduct: N-(2 cyan ethyl) valines, CEV), the data allow theoretical extrapolation to the initial protein adduct concentrations at the time of the accident (the MEV/CEV levels were from 1,616 pmol/g globin to 1,880 pmol/g globin and HEV/CEV levels from 1,407 pmol/g globin to 5,049 pmol/g globin, and correlated with inhaled 0.4-1.5 ppm ethylene oxide. These integrated, extrapolated internal doses, calculated on the basis of biological exposure equivalents, confirmed the clinical diagnosis for three patients, showing severe intoxication symptoms. Both, cmtDNA and 8-OHdG, as non-specific biomarkers of toxic effects, were elevated in four patients.

**Conclusion:**

The cases reported here, stress the importance of a suitable risk assessment and control measures. We put emphasis on the necessity of human biomonitoring guidelines and the urgency for the relevant limit values.

## Background

As a part of a global economy, an international network of suppliers, producers and retailers spans the world with logistics companies which connect the vast underlying commodities and the labor market [1]. Diverse interconnected companies and industries influence the logistics and transportation job market [1]. With the globalized production, most small and large companies import production parts, raw materials or goods from anywhere in the world. To ensure the preservation and quality of the goods, chemical agents for pest control have to be added to the goods or into the transport units. Thus a possible exposure to these toxic chemicals may occur not only for the applicators but also the receiver by off gassing from products, packing materials or transport units like containers. The international standards of the UN Food and Agriculture Organization (FAO) for phytosanitary measures (ISPM 15) require fumigation with methyl bromide (or heat treatment) of wooden packaging and flooring material (for pest control, i.e. spreading of alien species). Methyl bromide is a broad spectrum pesticide used in the agriculture for fighting a multitude of pests (e.g. used for pre-plant soil preparation), wood construction parts and for phytosanitary demands in shipping and storage [2–4]. Besides its desirable properties for pest control it also has ozone-depleting potency and was therefore banned in the Montreal Protocol in 2005. Nevertheless its usage is still allowed under the auspices of a critical use exemptions (CUE) wherever an adequate alternative is not available [3]. The fumigation of transport units or some agricultural applications is part of the CUEs. Due to the CUE more than 1,750 tons of methyl bromide were applied only in the agricultural sector of the State California in 2010 [5]. Additionally, other pesticides based on halo-alkenes, like ethylene dichloride, methyl chloride, propylene dichloride and chloropicrin are frequently used fumigants; small proportions are tainted with the methyl bromide substitutes sulphuryl difluoride, methyl iodide or ethylene oxide [6–8]. Several cases of intoxication after exposure to halogenated hydrocarbon pesticides, like methyl bromide or ethylene dichloride, have been observed. In a recent study methyl bromide exposure could be confirmed in 20% of the cases out of 164 patients with presumed intoxication from contact with halogenated hydrocarbon based fumigants [6]. Ambient-monitoring measurements performed on approx. 4,000 import containers in the harbors of Hamburg and Rotterdam revealed that less than three percent of the fumigated containers were labeled according to an international regulation [7–9]. Consequently, if no control measures are performed and the fumigation history is unknown, those dealing with fumigated items might be accidentally exposed and occasional intoxications may remain unrecognized. To some extent the symptoms caused by these fumigants are similar [3, 10–12]. Cases described in the literature show that the first clinical symptoms of methyl bromide intoxication may be unrecognized for up to three days resembling the flu (even in the most severe cases of intoxication with lethal outcome) [3, 13–15]. Exposure to the colorless, odorless, highly toxic, gaseous methyl bromide can occur through two primary routes: inhalation and dermal resorption [3]. Methyl bromide is neurotoxic and leads to damages in the central and peripheral nervous system, but also in the respiratory system, kidney, liver and eyes [3, 4]. Moreover some indications for a carcinogenic property of methyl bromide exist [3, 16–18].

Here, we report a series of three accidents of six storage room workers that occurred from 2010 to 2012; a follow up was performed in 2013. Though we are aware of many similar incidents across Europe [19], hardly any case is published in the scientific literature. On the other hand, insurance reports (confidential) or press releases document mysterious sudden illness by container unloading or even deaths on vessels, adding to uncertainty and calling for guidelines related to such accidences.

## Methods

### Case description

In a company, which received products from oversea, several employees developed signs of intoxication. The cases were submitted to one of us to consider a diagnosis of occupational disease and he escalated a toxicological supervision of the cases (Table 1). Six storage room workers were exposed to fumigants off gassing from shifted items in a medium sized European company importing electronic construction parts from overseas (Table 1). The exposures occurred three times within two years. The patients were working in a storage room. They were unloading ca. two overseas shipments a week at the time of the incidence. All patients worked 8 hours each working day in a storage room, where patients 1,3,4,5 and 6 unloaded the delivered items on a regular basis, unpacked wooden pellets with paper boxes covered with plastic (containing construction parts) and distributed the construction parts for the production line. Patient 2 supervised the working place. The delivered production parts were packed in several boxes on wooden pellets wrapped with plastic foils. The workers were responsible for unloading and distributing the delivered items into several separate areas on several different floors. After each incident the workers (patients 1, 3, 4, 5 and 6) noticed itchy skin, very red eyes and suffered from recurrent epistaxis, headaches and acrotaxia (intention tremor). After the second accident, four individuals (Pat. 1, 3, 4 and 5) additionally complained about paresthesia (pins and needles in the legs), dizziness, breathing difficulties and increasing irritability. After the third accident, patient 4 was on sick leave for several weeks; patients 1 and 3 developed immediate epistaxis with a severe headache.

**Table 1:** **Case description and demographic data**

**Accidental occurrence**	Six storage room workers were exposed to fumigants off gassing from shifted items in a medium sized European company importing electronic construction parts from Mexico and China. The exposures occurred three times (2010-2012), as reported by the patients. 48 hours after the first incidence a contracted industrial physician took blood samples and sent them to the clinical chemistry laboratory (Serum/EDTA blood, NaF blood) and sent samples to the commercial clinical chemistry laboratory to investigate intoxication parameters. Since no other information was provided to the laboratory, only a differential blood analysis was performed, no bromide,methyl bromide or other intoxication parameters were measured. As usual in such a case the blood samples were destroyed. Since two patients had persistent symptoms, the governmental industrial hygienist visited the company. He took air samples in the space between the delivered boxes; the laboratory analysis revealed 2.5-200 ppm residual methyl bromide. One year later, a second accident occurred involving the same employees. Ambient monitoring was performed, which showed the presence of methyl bromide and ethylene oxide in the air. Five days after the accident samples for biomonitoring (BM 2) were collected.
**The time line of the three accidents in the storage room**	The accidents occurred between 2010 and 2012. For the BM No. 1, the samples were taken 195 days after the first and 111 days after the second accident. For the BM No. 2, the biomonitoring samples were collected five days after the third accident; BM No 3. (follow up) was initiated 178 days after the third accident (2013).
**Exposure associated job description**	Exposed individuals: Patients 1-6 (aged: 32-54; 4 f, 2 m)
	Patient 1 (47, f); patient 2 (32,m); patient 3 (39, f); patient 4 (41,m); patient 5 (54, f); patient 6 (38, f).
	All patients worked 8 hours each working day in a storage space where patients 1,3,4,5 and 6 unloaded the delivered items on a regular basis, unpacked wooden pellets with paper boxes covered with plastic (containing with construction parts) and distributed the construction parts for the production line. Patient 2 supervised the working place.
**Exposures associated symptoms**	After each incident the workers (patients 1, 3, 4, 5 and 6) noticed itchy skin, very red eyes and suffered from recurrent epistaxis, headaches and acrotaxia. After the second accident four individuals (Pat. 1, 3, 4 and 5) additionally complained about paresthesia (pins and needles in the legs), dizziness, breathing difficulties and increasing irritability. After the third accident, patient 4 was on sick leave for several weeks; patients 1 and 3 developed immediate epistaxis with a severe headache.
**Description of the workplace**	Workplace: Medium sized company, which recently turned into a worldwide operating logistics unit with production and distribution centers, but no internal industrial hygiene unit. The 6 patients were working in a storage room. They were unloading ca. 2 overseas containers per week at the time of the incidence. According to the interviews the containers were not labeled for fumigants; No air monitoring was performed on a regular basis, though the workers sometimes perceived strange odors. The delivered production parts were packed in several boxes on wooden pellets wrapped with a plastic foil. The workers were responsible for unloading and distributing the delivered items into several separate areas on several different floors.

### Sampling

First accident: an industrial physician took blood samples 48 hours after the first incidence and five days after. Air samples were taken in the space between the delivered boxes. Three months later, a second accident occurred involving the same employees (Table 1). At this time blood samples were taken for a head-space analysis (whole blood); additional serum and hemoglobin were fractionated (for details see below). The samples were taken 111 days after the second accident. At the third incident (second biomonitoring), the samples were collected five days after the accident; the third biomonitoring (follow up) was initiated 178 days after the third accident. After the third incident, additional measurements of methyl mercapturic acid derivatives (MeMA) in urine were performed.

### Clinical examination of the participants

Patients (n = 6, aged from 32–54 yrs.) and control group (n = 30, with median age of 40 yrs.). Patients underwent standardized medical examination for the symptoms of fumigant intoxication as described earlier [10, 11, 19] and donated blood and urine samples. Both, patients and the control reference group, gave written permission. The study was approved by the local ethics committee (PV 3597 to XB/LTB). The clinical examination was indicative in the course of the social security case examination. The questionnaire (Fum-Ex2) is published [6] and available online at: http://eomsociety.org/attachments/FUM-EX%202_%20QUESTIONNAIRE_EOM%20ENGLISH%20VERSION.pdf. Fum-Ex 2 records time, duration and situation of exposure/s, applied protective devices and acute and chronic symptoms related/not related to exposure/s. For a complete diagnosis the job history is followed by a physical examination and functional tests of probably affected organs such as lung function testing, blood analyses, neurological and neuropsychological investigations [19], for details see below.

### Diagnostic scheme

- Occupational case history, FUM-EX2 questionnaire (headache, dizziness, concentration disorders, increased desire to sleep, emotional irritability, asthma, etc.)
- Lung function diagnostics (spirometry, methacholine challenge test, exercise test with blood gas analysis, CO diffusing capacity)
- Clinical Chemistry: Blood tests for liver enzymes, bromide and fluoride in serum and urine
- Biomonitoring (see part “human biomonitoring analysis” for details)
- Physical performance (grooved pegboard and finger tapping test)
- Olfactory test (scented sticks)
- Color vision test (Farnsworth)
- Specific neurological and neuropsychological tests:
  - ○ Test of crystallized and fluid intelligence (multiple choice-vocabulary-intelligence test, MWT-A, short test of general intelligence, KAI)
  - ○ Comprehensive neurological and neuropsychological tests covering orientation, information processing speed, selective attention/concentration tests, divided attention, sustained attention/vigilance, learning/memory tests, spatial-construction ability, logical thinking and abstraction, decision making and pre-morbid verbal intelligence
- Magnetic resonance tomography, MRT, and cranial computed tomography, c-CT, were done, if the before mentioned non-invasive diagnostic tests showed abnormal findings which may be due to cerebral lesions

Additionally we analyzed blood and urine samples of 30 healthy (smoking and non-smoking) control persons matching sex and age distribution to the patient group plus one additional sample from a heavy smoker (used as internal laboratory method control).

### Ambient monitoring

Five days after the first accident and after the third incident, air samples were taken in the storage room where the goods had been unpacked. These ambient monitoring measurements were conducted and performed by the local governmental industrial hygienist on duty. Methyl bromide and ethylene oxide were measured using the Selected Ion Flow Tube Mass Spectrometry method [8]. Additionally, several days after the third incident, air samples were also taken by the company’s engineer in the storage rooms. They were analyzed with a validated TD-GCMS method [20]. We have analyzed these air samples three times between the second and third accident in the same storage room and could not find any fumigant residues (data not shown). The samples were taken with an interval of ca. one month. The limitation however is that they were taken by the company’s engineer (only) in the middle of the closed storage facilities (not in the breathing zone).

### Human biomonitoring analyses

Fumigants and their derivatives were measured in blood samples (whole blood, serum) as follows: the substances were detected by gas chromatography/mass spectrometry (GCMS) (head space) in SIM mode in 60 m dimethylpolysiloxane DB1 capillary column under helium and temperature program 40–250°C. The analysis included the detection of methyl bromide (m/z 94, 79, 96), ethylene dichloride (m/z 62, 49, 98), methyl dichloride (m/z 84, 49, 86) ethylene oxide (m/z 29, 43), methyl iodine (m/z 142, 127 and chloropicrin/trichloro-nitro-methane (m/z 119, 117, 83) in blood. Methyl bromide could be detected at 0.19 μg/L (RT peak at 7.5 min) and quantitated at 0.56 μg/L and ethylene oxide (RT peak at 7.3 min) with a detection limit of 0.28 μg/L, a quantitation limit of 0.84 μg/L (as estimated with DIN 32645 method). Bromide ion was detected using the photometric method as described previously [21]. Long term biological exposure effects were determined by measuring reaction products (adducts) with macromolecules in blood, such as the hemoglobin (Hb) adducts N-methylvaline (MEV) and N-2-hydroxyethylvaline (HEV) as described previously [6]. The method is based on the N-alkyl Edman degradation [22, 23]. Due to controlling the influence of smoking on the Hb adducts levels, the Hb adduct N-2-cyanoethylvalin (CEV) was determined in addition to MEV and HEV by the procedure as well. Moreover, the current smoking status was objectivized by the determination of cotinine in urine using a GC-MS-method [24]. Creatinine was determined using a HPLC method described elsewhere [24]. Parallel to the sensitivity of the hemoglobin adduct, MEV-Assay was explored with the ROC (receiver operating characteristic) analysis, showing 70% sensitivity and 75% specificity by a cut off value of 483 pmol/g globin (AUC, Area Under Curve, values of 0.755) for the MEV measurement and 90% sensitivity and 35% specificity for the HEV analysis with a cut off value of 34 pmol/g globin (AUC = 0.550).

Mercapturic acids, derived from the chemical agents, were measured in urine using offline and online solid phase extraction of the analytes, respectively, with subsequent analytical separation and detection using LC-MS/MS as described elsewhere [25, 26]. 8-OHdG was determined with an enzyme-linked immunoabsorbent assay provided by JaICA’s [6]. The measurements of circulating mtDNA were described earlier [6]. All analytical methods were validated using inter- and extra laboratory quality assessment schemes from the German Society for Occupational and Environmental Medicine (G-EQUAS).

### Calculations of initial exposure levels and statistic

The elimination kinetics of the hemoglobin adduct level follows the equation y = -0.81 x (days post-exposure) + 95 within the first 120 days (= average life-span of erythrocytes) after exposure and with “y” as the percentage of the initial adduct concentration after a single exposure, as described by Bader [27, 28]. Assuming that we were dealing with individual, singular exposure, this equation allows us to approximate the theoretical initial ethylene oxide and methyl bromide levels at the time of exposure for the individual patients. Notably, the Commission for the Investigation of Health Hazards of Chemical Compounds in the Work Area of the Deutsche Forschungsgemeinschaft, DFG (German research council) provides equivalent values (EKA correlation) for carcinogenic substances. The EKA value correlates 1 ppm (1.83 mg/m3) ethylene oxide in the air with 90 μg HEV per L blood [29]. Based on these biological exposure equivalents, the smoking coefficients (for individual patients) and the elimination kinetics equation, we have calculated the theoretical individual exposure-associated MEV adduct values at the time of the exposure. All methods were routinely validated with control serum samples and additional standard set points (two analytic standards, one low and one high concentration were used as set points). T-tests were applied to calculate the distribution of the difference. The data analyses were performed with Graph PAD Prism Software 6.0 (Graph Pad Software Inc., San Diego, CA).

## Results

### Ambient monitoring

The workplace and the details on the incidence and sampling are described in Table 1. The air measurement verified a methyl bromide concentration from 2.5 to 200 ppm (mean: 125 ppm with 2.5-200 ppm min-max) in the storage room between the packing materials five days after the incidence. After the third accident, 15 ppm ethylene oxide could be detected in the storage room. The air samples were taken in the space between the packed electronic parts with trapped gas residues (in the breathing zone of the workers). Considering that both, ethylene oxide and methyl bromide, are extremely volatile, the concentrations of the fumigants in the air, on the day of the individual incidence, were presumable at least four to five times higher.

### Biological exposure monitoring

The fumigant levels in blood samples and the amounts of bromide ion in serum are shown in Table 2. We did not detect significantly elevated bromide levels in any of the analyzed serum samples. However, we detected low methyl bromide levels (0.24 μg/L) in the blood of patient 5 collected five days after the second incidence (BM2). No increased levels of either ethylene dichloride, chloropicrin, methyl iodine or dichloromethane were detected in the blood samples (all were < LOD). Patients 1, 3, 4, 5 and 6 showed slightly increased levels of ethylene oxide in blood and two patients displayed elevated methanol levels (Table 2).

**Table 2:** **Human biomonitoring performed after the second and third incident (BM1, BM2), for details see Table** 1

Pat.	Bromide [μg/L]		Methyl bromide [μg/L]		Ethylene oxide [μg/L]	Ethylene dichloride [μg/L]		Dichloro-methane [μg/L]		Methanol [mg/L]	
	BM1	BM2	BM1	BM2	BM2	BM2	BM2	BM1	BM2	BM1	BM2
**1**	2.8	**5.7**	<LOD	<LOD	**5.06**	<LOD	<LOD	<LOD	<LOD	<LOD	<LOD
**2**	2.5	**4.3**	<LOD	<LOD	<LOD	<LOD	<LOD	<LOD	<LOD	**5.73**	<LOD
**3**	2.8	**5.2**	<LOD	<LOD	**5.96**	<LOD	<LOD	0.51	<LOD	<LOD	<LOD
**4**	1.7	**4.8**	<LOD	<LOD	**3.00**	<LOD	<LOD	<LOD	**0.95**	<LOD	<LOD
**5**	**4.3**	**4.9**	<LOD	0.24^1^	**0.57**	<LOD	<LOD	<LOD	<LOD	<LOD	<LOD
**6**	3.1	**4.4**	<LOD	<LOD	**6.17**	<LOD	<LOD	<LOD	<LOD	0.67	<LOD
**Con.**		3.8	<LOD	<LOD	<LOD	<LOD	<LOD	<LOD	<LOD	<LOD	<LOD
**Ref.**		5	n.d.	n.d.	n.d.	n.d.	n.d.	n.d.	n.d.		

The data show measurements of bromide (serum) and methyl bromide, ethylene oxide, ethylene dichloride, dichloromethane and methanol in whole blood samples. The bold data, indicate for values higher than the references. For details see Methods.

Con. = controls (n = 30); n.d. not determined; Ref. DFG = reference values available from the Senate Commission of the German Research Council [DFG, 2012].

Slightly elevated levels of MeMA were detected in the urine sample of patient 1 only after the third incident (data not shown).

**Figure 1:**
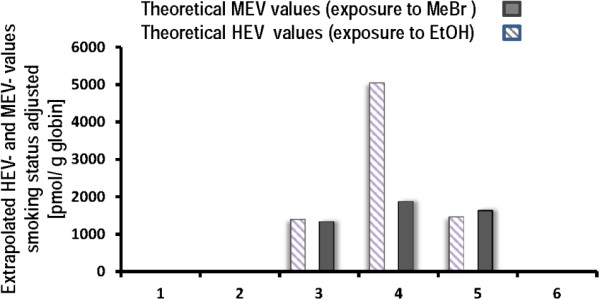
**Theoretical HEV and MEV hemoglobin adduct levels at the time of the individual exposure to ethylene oxide and methyl bromide, respectively.** All values were adjusted to smoking status using MEV/CEV and HEV/CEV coefficients. The data was modeled as described in the results part.

The results for MEV, HEV, CEV and cotinine are given in Table 3. The results for MEV and HEV were compared to reference values for the German general population considering the smoking status. The results revealed elevated levels of MEV and HEV for patient 4 by the factor of 1.9 and 2.3, respectively, for patient 5 by the factor of 1.7 and 1.4, respectively, and for patient 3 by the factor of 1.4 (MEV). Patient 5 had remarkably high hemoglobin adduct levels at BM 2. Moreover, patient 3 had higher MEV and HEV levels in BM 3. Patients 1, 2 and 6 were in the expected range of smoker or non-smoker at the time of sampling (shown in Table 3). We have calculated (for details see Methods) the theoretical individual exposure-associated (Figure 1) MEV adduct values at the time of the exposure with 1,616 pmol/ g globin for patient 3, 1,880 pmol/g globin for patient 4 and 1,640 pmol for patient 5 (after the methyl bromide exposure). The respective HEV values were: 1,403 pmol/ g globin for patient 3 (which correlate with inhaled 0.4 ppm ethylene oxide); for patient 4 5,049 pmol/globin (correlating with 1.5 ppm inhaled ethylene oxide) for patient 4 and 1,467 pmol/ g globin (correlating with 0.4 pmol ethylene oxide in the air) for patient 5.

**Table 3:** **Biomonitoring for hemoglobin adducts MEV, HEV and CEV as well as for cotinine: BM1; BM2, BM3**

Pat.	MEV [pmol/g globin]			HEV [pmol/g globin]			CEV [pmol/g globin]			Cotinine [μg/g Crea.]		
	BM1	BM2	BM3	BM1	BM2	BM3	BM1	BM2	BM3	BM1	BM2	BM3
**1**	453	447	-^2^	26	46	-^2^	0^1^	0^1^	-^2^	1	0^1^	-^2^
**2**	**485**	**476**	-^2^	36	34	-^2^	0^1^	0^1^	-^2^	2	8	-^2^
**3**	**546**	388	-^2^	132	54	-^2^	70	31	-^2^	1469	1169	-^2^
**4**	**726**	**652**	**656**	**350**	**348**	**509**	162	150	245	5049	3894	2902
**5**	518	**653**	**609**	140	**212**	**266**	26	62	91	196	1182	1390
**6**	450	459	-^2^	115	107	-^2^	48	46	-^2^	1404	1409	-^2^
**Con.**	NS 452			NS 43			NS <2			NS < 10/		
	S 539			S 132			S150			S >100		
Ref.	NS 320			NS < 75			NS <20			NS < 10/		
	S 390			S 150			S 150			S >100		

^1^under limit of detection, ^2^samples not provided. The bold data, indicate for values higher than the references. For details see Methods.

Con. = mean controls values (n = 30); n.d. not determined; Ref. = mean DFG reference values available from the Senate Commission of the German Research Council [DFG, 2012]. NS = non smoker, S = smoker.

### Biological effect monitoring

After the third incident, biological effect biomarkers were additionally measured (Table 4). Increased 8-OHdG levels (as a short term marker for oxidative stress) were observed in urine samples of patient 3 (142% increase vs. the references), patient 4 (147% increased levels) and in patient 5 (207% increase). When measuring the levels of the circulating mitochondrial DNA, as a non-specific effect toxicity biomarker [6], elevated levels could be detected in all patients. Moreover, patients 3 and 5 had the strongest increase in the levels of the circulating mitochondrial DNA in serum after the third intoxication (Table 4).

**Table 4:** **Additional effect biomonitoring performed after the third incident**

Patient	8-OHdG (urine) [μg/g Crea.]	mtDNA79 (serum) [arbitrary]	Creatinine (urine) [g/L]
Pat.1	**6.2**	**32.3**	2.42
Pat.2	4.6	**14.5**	0.40
Pat.3	**9.8**	**796.5**	1.34
Pat.4	**10.2**	**26.2**	1.35
Pat.5	**14.3**	**147.8**	1.43
Pat.6	**12.9**	**86.9**	0.41
Con.	6.7	0.08	
Ref.	n.d.	n.d.	0.5-3.0

Human biomonitoring (BM2) measurements were performed in samples collected five days after the third accident. The data show measurements of 8-OHdG as well as the circulating mitochondrial DNA (mtDNA79). The bold data, indicate for values higher than the references.

The controls (con.) represent median values from 30 non-exposed individuals measured in the same analytical step as the patient samples.

n.d. not determined; Ref. = DFG = reference values available from the Senate Commission of the German Research Council [DFG, 2012].

## Discussion

Here, we report a series of accidents of six storage room workers in a medium sized European company regularly importing electronic production parts from abroad. Three out of four persons with presumed exposure showed serious intoxication symptoms with itchy skin, very red eyes, dizziness, breathing difficulties and increasing irritability. They suffered from recurrent epistaxis, headaches and acrotaxia (intension tremor) with paresthesia (pins and needles in the legs) and developed immediate epistaxis with a severe headache. Since the ambient monitoring of air samples was taken five days after the accident, the initial fumigant concentration in the air at the time of the incident was presumably at least four to five times higher than the measured values. A correct strategy for the human biomonitoring sampling time in the reported cases should be one to three days post exposure, in order to detect fumigant levels in blood (biological exposure monitoring). Unfortunately, this was not the case, besides a discrete enhancement of ethylene oxide levels in some patients. Given the published half-life of bromide in serum with 10–12 days ca. 0.25% should be cleared after five days (BM2). Though our reference group has median bromide levels of 3.8 μg/L and our internal method-lab controls (n = 50 unexposed) show a median value of 3.1 μg/L, the values near the background levels do not allow any extrapolation. We detected a low methyl bromide level (0.24 μg/L) in the blood of patient 5, collected five days after the second incidence (BM2), which might indicate the incorporation at the day of the incidence. Only one study shows human kinetics data on methyl bromide levels in blood, with the elimination kinetics for up to 21 days after a fatal accident [15]. Little human literature data indicate that both, methyl bromide and ethylene oxide, have longer half lives in humans than in experimental animals with 12–99 h for ethylene oxide and up to 11 days for methyl bromide (depending on concentration, exposure circumstances and genetic predispositions) [15, 30, 31].

No reference values exist for methyl bromide (however, since the data from the control groups show values below the limit of detection, we assume that there is no general background contamination).Though there is a weak indication for the presence of fumigants in blood (2 patients), these exposure biomonitoring data do not allow any firm interpretation The half-life of mercapturic acids, MeMA is with 12–48 h extremely short, only allowing the detection of immediate exposure/s.

In-vivo dose monitoring by means of adducts to macromolecules after exposure to methylating agents has been well characterized earlier [23]. Ethylene oxide is known to alkylate (2-hydroxylate) cellular macromolecules including proteins and DNA. Methyl bromide is an established methylating agent. The hemoglobin adducts methyl valine and hydroxyl ethyl valine are biochemical effect markers, elevated after exposure to alkylating chemicals like methyl bromide or ethylene oxide. MEV and HEV in particular are adducts of methylating agents formed on the N-terminal valine of hemoglobin [32, 33]. Hemoglobin adducts: N- (2-hydroxyl ethyl valine), HEV and 2-methylvaline, MEV have been used as a molecular dosimeter for exposure to ethylene oxide and methylating agents, respectively [34]. The monitoring of hemoglobin adducts additionally allows to monitor past exposure and to generate extrapolated data that might improve the risk assessment strategy. Bader and his colleagues [27, 28] proved that hemoglobin adduct levels can be used for monitoring single accidental exposures in case of an industrial accident. They provided a mathematical model allowing the extrapolation of the data. We hereby calculated the theoretical exposure/s to methyl bromide and ethylene oxide at the time of the individual incidents. Based on this data, the intoxication could be confirmed for three patients out of six (patients, 3, 4 and 5); data for patients 1, 2 and 6 did not allow any extrapolation. Considering the suggested German acceptance limit values with 0.1 ppm and the tolerance limit values of 1 ppm for ethylene oxide, it appears that the patients 3, 4 and 5 were exposed to fumigant levels higher than the acceptable values (0. 1 ppm). An enhancement of the tolerable exposure level (1 ppm) could be extrapolated for patient 4. Currently no biological equivalent values are available for methyl bromide. Considering the current limit values of 1 ppm for methyl bromide, we could presume the exposure/s above the tolerable fumigant levels for all three patients 3, 4 and 5. The clinical examination data from these patients (data not shown here) also support the evidence of intoxication. No intoxication could be confirmed for patients 1, 2 and 6. Patient 2 was supervising the rest of the crew and patients 1and 6 were mostly engaged in other areas of the storage room after the first incidence having less direct contact with (newly delivered) unloaded products. Notably, after the first accident the company reorganized the storage area with a separate zone for unpacking the incoming shipment. As reported, the patients showed serious intoxication symptoms with itchy skin, very red eyes, dizziness, breathing difficulties and increasing irritability. They suffered from recurrent epistaxis, headache, acrotaxia, (intention tremor) paresthesia, and/or developed immediate epistaxis. It is important to note that the symptoms of acute methyl bromide intoxication comprise irritation of mucosa, nausea, headache, vision disorders, apraxia and acrotaxia [10, 11, 19], neuropathy [13], encephalopathy [35, 36], paresthesia [37] or even death [14, 15].

Our case studies indicate a need for an early and comprehensive prevention system including dedicated rules for the occupational hygiene and biomonitoring procedures. Most of the transport units (either unopened container or plastic covered pellets) are commonly unpacked outside the harbor areas and not at the border. Intoxication incidents reported herewith occur in many small European companies [19] and can presumably happen in any country of the globalized world.

### Limitations

There are several limitations. First of all, the calculation performed was based on the assumption that we are dealing with single exposures as reported to the governmental industrial physician and the insurance company. The intermediate exposure/s (between the reported accidents) is likely causing cumulative accumulation of adducts in blood. Markedly, we could not detect fumigants on three randomly chosen control air measurements (data not shown). Further limitations are given by the smoking habits of the patients: we had to consider individual adduct levels caused by smoking.

Though the measurement of adducts as a biomarker of exposure is several orders of magnitude more sensitive than disease epidemiological investigations [38], an adduct formation from endogenously produced ethane with S-adenosylmethionine as the reactive product have been documented [23, 39]. To a small extent, ethane present in urban pollution can enhance basal HEV levels [39]. Variations of MEV and HEV levels are partly hereditary especially with respect to a glutathione S-transferase catalyzed metabolism [40], while suggested dietary meat consumption has no effect on MEV or HEV levels [41]. Törnqvist and her colleagues have documented background (non-occupational) adduct levels with 16 [9–38] pmol/g globin for HEV and 225 [217–372] pmol/g globin for MEV, in non-smoking subjects [22, 40]. However, it has to be mentioned that hemoglobin adduct levels need several weeks for reaching steady-state. Thus, only high accidental exposure can produce a detectable impact on Hb adduct levels.

The biological effect markers mitochondrial DNA and 8-OhDG confirm possible past intoxication with toxic/ carcinogenic chemicals, however provide no additional information related to individual incidents. Notably, patients 3, 4 and 5 showed persistent neurological symptoms one year after the incidents. Possible long term effects should not be underestimated, since ethylene oxide and methyl bromide are presumably carcinogenic to humans [3, 34].

## Conclusions

The data emphasize that there is a lack of knowledge on risk assessment and monitoring strategies for incident scenarios. The cases reported here, stress the importance of the human biomonitoring guidelines for occasional exposure to toxic substances. They should provide necessary information to the industrial physicians on how to choose the required biomarker, the adequate matrix and the sampling time. The guidelines must put a strong emphasis on an immediate contact with a qualified laboratory directly after the intoxication to get adequate biomonitoring support. At workplaces in the country sites, sufficient awareness is still needed on possible risks from fumigants. Unfamiliarity with possible risks due to gas emissions leave many persisting intoxication cases unrecognized.

The international regulatory bodies should reconsider if there is a phytosanitary need for fumigation of transport units containing electronic construction parts.

## Abbreviations

CUE: Critical use exemptions
FAO: UN Food and Agriculture Organization
HEV: N-(2-hydroxy ethyl) valine
CEV: N-2-cyanoethylvaline
cmtDNA: Circulating mitochondrial DNA
MeMa: Methyl mercapturic acid derivatives
MEV: N-methyl valine.

## References

[1] Wible B, Mervis J, Wigginton NS (2014). The global supply chain. Rethinking the global supply chain. Introduction. Science.

[2] FAO (2007). Food and Agriculture Organization of the United Nations, International standards for phytosanitary measures. Guidelines for Regulating Wood Packaging Material in International Trade.

[3] Budnik LT, Kloth S, Velasco-Garrido M, Baur X (2012). Prostate cancer and toxicity from critical use exemptions of methyl bromide: environmental protection helps protect against human health risks. Environ Health.

[4] **Methyl Bromide, CAS No. 74–83–9.**. http://www.cdc.gov/niosh/npg/npgd0400.html

[5] Barrett JR (2013). Getting the drift: methyl bromide application and adverse birth outcomes in an agricultural area. Environ Health Perspect.

[6] Budnik LT, Kloth S, Baur X, Preisser AM, Schwarzenbach H (2013). Circulating mitochondrial DNA as biomarker linking environmental chemical exposure to early preclinical lesions elevation of mtDNA in human serum after exposure to carcinogenic halo-alkane-based pesticides. PLoS One.

[7] Budnik LT, Fahrenholtz S, Kloth S, Baur X (2010). Halogenated hydrocarbon pesticides and other volatile organic contaminants provide analytical challenges in global trading. J Environ Monit.

[8] Baur X, Poschadel B, Budnik LT (2010). High frequency of fumigants and other toxic gases in imported freight containers–an underestimated occupational and community health risk. Occup Environ Med.

[9] Fahrenholtz S, Huhnerfuss H, Baur X, Budnik LT (2010). Determination of phosphine and other fumigants in air samples by thermal desorption and 2D heart-cutting gas chromatography with synchronous SIM/Scan mass spectrometry and flame photometric detection. J Chromatogr A.

[10] Preisser AM, Budnik LT, Hampel E, Baur X (2011). Surprises perilous: Toxic health hazards for employees unloading fumigated shipping containers. Sci Total Environ.

[11] Preisser AM, Budnik LT, Baur X (2012). Health effects due to fumigated freight containers and goods: how to detect, how to act. Int Marit Health.

[12] Preisser A, Poppe A, Budnik LT, Baur X (2007). Intoxikationen beim entladen von import-containern in einer maschinenfabrik. Zbl Arbeitsmed.

[13] Lifshitz M, Gavrilov V (2000). Central nervous system toxicity and early peripheral neuropathy following dermal exposure to methyl bromide. J Toxicol Clin Toxicol.

[14] Langard S, Rognum T, Flotterod O, Skaug V (1996). Fatal accident resulting from methyl bromide poisoning after fumigation of a neighbouring house; leakage through sewage pipes. J Appl Toxicol.

[15] Horowitz BZ, Albertson TE, O’Malley M, Swenson EJ (1998). An unusual exposure to methyl bromide leading to fatality. J Toxicol Clin Toxicol.

[16] Alavanja MC, Samanic C, Dosemeci M, Lubin J, Tarone R, Lynch CF, Knott C, Thomas K, Hoppin JA, Barker J, Coble J, Sandler DP, Blair A (2003). Use of agricultural pesticides and prostate cancer risk in the Agricultural Health Study cohort. Am J Epidemiol.

[17] Alavanja MC, Bonner MR (2012). Occupational pesticide exposures and cancer risk: a review. J Toxicol Environ Health B Crit Rev.

[18] Barry KH, Koutros S, Lubin JH, Coble JB, Barone-Adesi F, Beane Freeman LE, Sandler DP, Hoppin JA, Ma X, Zheng T, Alavanja MC (2012). Methyl bromide exposure and cancer risk in the Agricultural Health Study. Cancer Causes Control.

[19] Baur X, Horneland A-M, Fischer A, Stahlmann R, Budnik LT (2014). How to handle import containers safely. Int Marit Health.

[20] Fahrenholtz S, Hühnerfuss H, Baur X, Budnik LT (2011). Messung von Phosphorwasserstoff neben flüchtigen organischen Substanzen mittels Gaschromatographie. Zbl Arbeitsmed.

[21] Müller M, Reinhold P, Lange M, Zeise M, Jurgens U, Hallier E (1999). Photometric determination of human serum bromide levels–a convenient biomonitoring parameter for methyl bromide exposure. Toxicol Lett.

[22] Van Sittert NJ, Abgerer J, Bader M, Blaszkewicz M, Ellrich D, Krämer A, Lewanlter J, Angerer J, Schaller KH (1997). N-2-Cyanoethylvaline, N-2-Hydroxyethylvaline, N-methylvaline (as evidence of exposure to acrylonitrile, ethylene oxide as well as methylating agents). Analyses of Hazardous Substances in Biological Materials.

[23] Törnqvist M, Kautiainen A (1993). Adducted proteins for identification of endogenous electrophiles. Environ Health Perspect.

[24] Heinrich-Ramm R, Wegner R, Garde AH, Baur X (2002). Cotinine excretion (tobacco smoke biomarker) of smokers and non-smokers: comparison of GC/MS and RIA results. Int J Hyg Environ Health.

[25] Eckert E, Drexler H, Göen T (2010). Determination of six hydroxyalkyl mercapturic acids in human urine using hydrophilic interaction liquid chromatography with tandem mass spectrometry (HILIC-ESI-MS/MS). J Chromatogr B Analyt Technol Biomed Life Sci.

[26] Eckert E, Göen T (2014). Rapid determination of four short-chain alkyl mercapturic acids in human urine by column-switching liquid chromatography-tandem mass spectrometry. J Chromatogr B Analyt Technol Biomed Life Sci.

[27] Bader M, Will W, Frey G, Nasterlack M (2012). Analysis of protein adducts as biomarkers of short-term exposure to ethylene oxide and results of follow-up biomonitoring. Arh Hig Rada Toksikol.

[28] Bader M, Will W, Rossbach B, Triebig G, Drexler H, Letzel S, Nowak D (2012). Follow-up-Biomonitoring nach akuter Exposition. Biomonitoring in Arbeitsmedizin und Umweltmedizin - Orientierungshilfe für Betrieb, Praxis und Klinik.

[29] Deutsche Forschungsgemeinschaft DFG (2014). List of MAK and BAT Values 2014. Commission for the Investigation of Health Hazards of Chemical Compounds in the Work Area. Report No. 50.

[30] Li Q, Csanady GA, Kessler W, Klein D, Pankratz H, Putz C, Richter N, Filser JG (2011). Kinetics of ethylene and ethylene oxide in subcellular fractions of lungs and livers of male B6C3F1 mice and male fischer 344 rats and of human livers. Toxicol Sci.

[31] Csanady GA, Denk B, Putz C, Kreuzer PE, Kessler W, Baur C, Gargas ML, Filser JG (2000). A physiological toxicokinetic model for exogenous and endogenous ethylene and ethylene oxide in rat, mouse, and human: formation of 2-hydroxyethyl adducts with hemoglobin and DNA. Toxicol Appl Pharmacol.

[32] Bolt HM, Gansewendt B (1993). Mechanisms of carcinogenicity of methyl halides. Crit Rev Toxicol.

[33] Bolt HM, Leutbecher M (1993). Dose-DNA adduct relationship for ethylene oxide. Arch Toxicol.

[34] IARC, Lyon (1994). Ethylene oxide. IARC Monographs on the Evaluation of Carcinogenic Risks in Humans: Some Industrial Chemicals.

[35] Kim EA, Kang SK (2010). Occupational neurological disorders in Korea. J Korean Med Sci.

[36] Park HJ, Lee KM, Nam JK, Park NC (2005). A case of erectile dysfunction associated with chronic methyl bromide intoxication. Int J Impot Res.

[37] De Haro L, Gastaut JL, Jouglard J, Renacco E (1997). Central and peripheral neurotoxic effects of chronic methyl bromide intoxication. J Toxicol Clin Toxicol.

[38] Törnqvist M, Ehrenberg L (1994). On cancer risk estimation of urban air pollution. Environ Health Perspect.

[39] Törnqvist M (1994). Is ambient ethene a cancer risk factor?. Environ Health Perspect.

[40] Törnqvist M, Svartengren M, Ericsson CH (1992). Methylations in hemoglobin from monozygotic twins discordant for cigarette smoking: hereditary and tobacco-related factors. Chem Biol Interact.

[41] Gurney JG, Chen M, Skluzacek MC, Kasum CM, Carmella SG, Villalta PW, Hecht SS (2002). Null association between frequency of cured meat consumption and methylvaline and ethylvaline hemoglobin adduct levels: the N-nitroso brain cancer hypothesis. Cancer Epidemiol Biomarkers Prev.

